# Clinical characteristics of ulcerative colitis patients with different types of *Helicobacter pylori* infection

**DOI:** 10.1128/spectrum.03554-23

**Published:** 2024-04-15

**Authors:** Weidong Liu, Qi Jiang, Shenglong Xue, Wenjia Hui, Wenjie Kong, Mengxia Zhang, Feng Gao

**Affiliations:** 1 College of Life Science and Technology, Xinjiang University, Urumqi, China; 2 Department of Gastroenterology, People’s Hospital of Xinjiang Uygur Autonomous Region, Urumqi, China; 3 Xinjiang Clinical Research Center for Digestive Diseases, Urumqi, China; University of Brescia, Brescia, Italy

**Keywords:** ulcerative colitis, *Helicobacter pylori*, virulence factors, hematology index, extraintestinal manifestations

## Abstract

There has been a suggestion of a potential protective effect of *Helicobacter pylori* (*H. pylori*) in the development of ulcerative colitis (UC). Virulence factor is an important factor in *H. pylori*, but little is known about the clinical characteristics of ulcerative colitis. In this retrospective study, a total of 322 patients with UC were analyzed. They were divided into three groups based on *H. pylori* antibody typing classification: type I *H. pylori* infection group, type II *H. pylori* infection group, and *H. pylori*-negative group. The study aimed to analyze the clinical characteristics of different types of *H. pylori* infection groups. The proportions of disease course, nationality, clinical type, and disease severity among UC patients in different types of *H. pylori* infection groups exhibited statistically significant differences (*P* < 0.05). However, no significant differences were observed in terms of sex, age, smoking status, alcohol consumption, body mass index (BMI), or lesion range (*P* > 0.05). Among the extraintestinal manifestations, the incidence of joint lesions in the type I *H. pylori* infection group was significantly lower compared with *H. pylori*-negative group (*P* < 0.05). The levels of red blood cell, hemoglobin, packed cell volume, albumin, A/G, and alanine aminotransferase were significantly higher in the type I *H. pylori* infection group compared with both the type II *H. pylori* infection group and *H. pylori*-negative group in the hematology index. Conversely, the levels of D-Dimer, C-reactive protein, and erythrocyte sedimentation rate were significantly lower in the type II *H. pylori* infection group (*P* < 0.05). In patients with UC, infections with the highly virulent type I *H. pylori* exhibit a negative correlation with both the severity of the disease and extraintestinal manifestations. While infections with the less virulent type II *H. pylori* are negatively correlated only with the disease severity. Therefore, the virulence factors of *H. pylori* play an important role in the regulation of UC.

**IMPORTANCE:**

The number of patients with ulcerative colitis (UC) has increased dramatically worldwide, posing a global public health challenge, There has been a suggestion of a potential protective effect of *Helicobacter pylori* in the development of UC. Virulence factor is an important factor in *H. pylori*, but high-quality clinical evidence is lacking. This study comprehensively analyzed the clinical characteristics of UC patients with different types of *H. pylori* infection. Infections with the highly virulent type I *H. pylori* are found to be negatively correlated with the severity of the disease as well as extraintestinal manifestations, whereas infections with the less virulent type II *H. pylori* demonstrate a negative correlation solely with disease severity. These results suggest that the virulence factors of *H. pylori* play a pivotal role in UC. Consequently, virulence factors should be taken into consideration when targeting *H. pylori* eradication in clinical practice, particularly in UC patients. It is crucial to evaluate the individual benefits to optimize personalized eradication therapies.

## INTRODUCTION

Ulcerative colitis (UC) is a chronic and recurrent inflammatory bowel disease characterized by intestinal inflammation and epithelial damage, affecting a substantial number of patients. The global incidence of UC cases has been experiencing a rapid surge, presenting formidable challenges to the field of global public health (1). The pathogenesis of UC involves multiple factors including genetic susceptibility, environmental factors, immune response dysregulation, and the interaction between microbiota and host (2). *Helicobacter pylori* infection has been found to have a negative correlation with the prevalence of UC, implying a potential protective effect against the onset of this disease (3, 4). However, the beneficial effects of eradicating *H. pylori* in the context of inflammatory bowel diseases, gastroesophageal reflux disease, asthma, and other diseases remain a subject of ongoing controversy (5). The genetically diverse nature of *H. pylori* gives rise to significant variations in virulence among different strains, which are closely associated with gastrointestinal diseases. The virulence factors of the pathogen not only elicit inflammatory responses but also control and regulate these responses to maintain chronic inflammation (6). Furthermore, bacterial virulence factors are crucial for host-microecological interaction (7). In clinical practice, *H. pylori* antibody typing enables the classification of *H. pylori* into type I (CagA^+^/VacA^+^) or type II (CagA^-^/VacA^-^) based on their respective expression levels of cytotoxin-associated gene A protein (CagA) and vacuolating toxin A protein (VacA). Our previous studies have demonstrated that type I *H. pylori*, characterized by higher virulence factors and migratory ability, may contribute to the progression of gastric mucosal atrophy, while type II *H. pylori* with lower virulence may hold greater clinical utility value (8). Therefore, it is crucial to investigate the protective effects of *H. pylori* on ulcerative colitis from the perspective of its virulence factors.

## MATERIALS AND METHODS

### Sampling subjects

A retrospective analysis included a cohort of 322 patients with UC in People’s Hospital of Xinjiang Uygur Autonomous Region, admitted between January 2015 and June 2023. Inclusion criteria: All patients fulfilled the diagnostic criteria for ulcerative colitis as outlined in Chinese consensus on diagnosis and treatment of inflammatory bowel disease (Beijing, 2018) (9). Patients with incomplete hematology index, and patients with a prior history of colorectal surgery. All patients were admitted for the first time, and the hematological indices reflect the initial measurement results. Exclusion criteria: Recurrent hospitalization of patients with UC, patients with inconsistent results in ^14^C-urea breath test, and *H. pylori* antibody typing classification. Based on *H. pylori* antibody typing, 322 UC patients were categorized into three groups: type I *H. pylori* infection (91 patients), type II *H. pylori* infection (58 patients), and *H. pylori*-negative group (173 patients). This study was approved by the Ethics Committee of People’s Hospital of Xinjiang Uygur Autonomous Region (KY202306173).

### Hematology index

Relevant hematology indices were obtained by performing blood routine and blood biochemical index on patients diagnosed with UC. This study included 27 major hematology indices: red blood cell (RBC), white blood cell (WBC), hemoglobin (HGB), platelet count (PLT), packed cell volume (PCV), neutrophil (NE), lymphocyte (LY), monocyte (MO), eosinophil (EO), basophil (BA), total bilirubin (TBIL), direct bilirubin (DBIL), indirect bilirubin (IBIL), protein total (TP), albumin (ALB), globulin (GLO), A/G, alanine aminotransferase (ALT), aspartate aminotransferase (AST), AST/ALT, potassium (K), sodium (Na), chloride (CL), D-dimer, C-reactive protein (CRP), erythrocyte sedimentation rate (ESR), and hypersensitive C-reactive protein (hs-CRP).

### ^14^C-urea breath test

Patients with UC were instructed to ingest a ^14^C urea capsule (Shenzhen Zhonghe Headway Bio-Sci Tech Co., Led.) on an empty stomach or 2 h after meals, followed by a seated position for 25 min. Subsequently, they were required to exhale into the gas collector for approximately 3 min until the liquid indicator color faded. Afterward, 4.5 mL of scintillation solution was added to the gas collector and gently inverted three times for thorough mixing prior to being transferred to the *H. pylori* detector (HUBT01) for rapid detection, with a required time of 1 min. The sample was classified as positive for *H. pylori* infection if the detection value was ≥100 dpm, and negative if the detection value was <100 dpm.

### *H. pylori* antibody typing classification

The venous blood samples of patients with ulcerative colitis were collected in volumes of 2–3 mL. The serum was subsequently obtained by centrifugation at 3,500 rpm for 10 min. The presence of antibodies against *H. pylori* was detected using western blot analysis. The *H. pylori* antibody typing classification kit (western blot) was provided by Shenzhen Blot Biological Products, Shenzhen, China, Co., Ltd. The qualitative comparison between the imprinting membrane strip and the standard strip was conducted following serum antibody binding, enzyme-linked reaction, color development, and termination of the reaction. Positive results for type I *H. pylori* infection were indicated by the simultaneous presence of either or both the CagA and VacA zones. Positive results for type II *H. pylori* infection were characterized by the presence of either or both urease A (UreA) and urease B (UreB) zones, without the detection of the CagA or VacA zone. Negative results were defined as the absence of any positive zone except the quality control.

### Statistical analysis

The data sorting and statistical analyses were performed using SPSS 25.0 software. Categorical data were expressed as *n* (%) and compared between groups using either the *χ*^*2*^ test or Fisher’s exact test as appropriate. Continuous data following a normal distribution were presented as the mean ± standard deviation. Multi-group comparisons for such continuous data were conducted through analysis of variance (ANOVA), and if a statistically significant difference was found, the least significant difference (LSD) method was used for pairwise comparisons, assuming equal variances. Continuous data not following a normal distribution were presented as the median and interquartile range. Non-parametric tests such as the Kruskal-Wallis test for multi-group comparisons or the Mann-Whitney U test for pairwise comparisons were employed when data did not meet the assumptions of normality or equal variances. Statistical significance was established at *P* < 0.05.

## RESULTS

### General data

Among the 322 patients with UC, there were 166 males and 156 females, resulting in a male-to-female ratio of 1.06. There were 143 Uyghur individuals, 172 Han individuals, and 7 individuals from other nationalities. The mean age was 51.5 ± 13.7 years. According to the *H. pylori* antibody typing classification, there were 91 cases in type I *H. pylori* infection group, 58 cases in type II *H. pylori* infection group, and 173 cases in *H. pylori*-negative group. The three groups did not exhibit any significant differences in terms of gender, age, body mass index (BMI), smoking, drinking, and lesion range (*P* > 0.05). However, statistically significant differences were observed in disease course, nationality, clinical type, and disease severity (*P* < 0.05) ( Table 1). In terms of disease severity, the proportion of mild disease in the type I *H. pylori* infection group was higher than that in the type II *H. pylori* infection group and the *H. pylori*-negative group, and the difference was statistically significant (*χ*^*2*^ = 5.787; 9.316, *P* < 0.05).

**TABLE 1:** General clinical features of UC with different types of *H. pylori* infection

	Total (*n* = 322)	Type I *H*. *pylori* infection group (*n* = 91）	Type II *H*. *pylori* infection group (*n* = 58）	*H. pylori*-negative group (*n* = 173）	Statistical value (*χ^2^/F*）	*P* value^*a*^
Gender						
Male	166 (51.6%)	45 (49.5%)	25 (43.1%）	96 (55.5%)	2.884	0.236
Female	156 (48.4%)	46 (50.5%)	33 (56.9%）	77 (44.5%)		
M/F	1.06	0.98	0.76	1.25		
Age (years)	51.5 ± 13.7	51.3 ± 13	51 ± 12	51.8 ± 14.7	0.097	0.907
BMI	22.9 ± 3.7	23.54 ± 4.04	22.15 ± 3.79	22.87 ± 3.45	2.552	0.080
Disease course (Month)	58.2 (3,84)	24 (3,72)	9.5 (2,48)	36 (6,108)	10.005	0.007*
Smoking	56 (17.4%)	19 (20.9%)	8 (13.8%)	29 (16.8%)	1.337	0.513
Drinking	50 (15.5%)	15 (16.5%)	7 (12.1%)	28 (16.2%)	0.547	0.761
Nationality						
Han	172 (53.4%)	35 (38.5%）	34 (58.6%）	103 (59.5%）	11.777	0.019*
Uyghur	143 (44.4%)	54 (59.3%）	23 (39.7%）	66 (38.2%）		
Others	7 (2.2%)	2 (2.2%）	1 (1.7%）	4 (3.3%）		
Clinical type						
primary type	108 (33.5%)	32 (35.2%）	28 (48.3%）	48 (27.7%）	8.338	0.015*
Chronic recurrent type	214 (66.5%)	59 (64.8%）	30 (51.7%）	125 (72.2%）		
Disease severity						
Mild	163 (50.6%)	59 (64.8%）	26 (44.8%）	78 (45.1%）	17.978	0.000*
Moderate	112 (34.8%)	28 (30.8%）	25 (43.1%）	59 (34.1%）		
Severe	47 (14.6%)	4 (4.4%）	7 (12.1%）	36 (20.8%）		
Lesion range						
Colon	65 (20.2%)	24 (26.4%）	11 (19%）	30 (17.3%）	5.595	0.231
Left hemicolon	125 (38.8%)	38 (41.8%）	23 (39.7%）	64 (37%）		
Extensive colon	132 (41%)	29 (31.8%）	24 (41.3%）	79 (45.7%）		

^*a*^ Comparison between the three groups, *P<0.05.

### Analysis of extraintestinal manifestations and complications of UC with different types of *H. pylori* infection

Among the 322 patients with UC, the major extraintestinal manifestations included joint lesions (7.8%), cutaneous mucosal manifestations (4%), ocular lesions (1.2%), liver and biliary diseases (20.8%), and thromboembolic diseases (1.2%). The main complications observed included intestinal perforation (1.6%), intraepithelial neoplasia (2.5%), and canceration (0.6%). Compared with the different types of *H. pylori* infection groups, the proportions of cutaneous mucosal manifestations, and liver and bile diseases in type I *H. pylori* infection group were lower than those in type II *H. pylori* infection group and *H. pylori*-negative group, but there was no statistical significance (*P* > 0.05). While, the incidence of joint lesions in type I *H. pylori* infection group was significantly lower than that in *H. pylori*-negative group (*χ*^2^ = 6.285, *P* < 0.05) (Table 2).

**TABLE 2:** Extrenteral manifestations and complications of UC with different types of *H. pylori* infection

	Total (*n* = 322)	Type I *H*. *pylori* infection group (*n* = 91）	Type II *H*. *pylori* infection group (*n* = 58）	*H. pylori*-negative group (*n* = 173）	Statistical value (*χ^2^*）	*P* value^*a*^
Extraintestinal manifestation						
Joint lesions	25 (7.8%)	2 (2.2%）	4 (6.9%）	19 (3.7%）	5.454	0.023*
Cutaneous mucosal manifestations	13 (4%)	3 (3.3%）	3 (5.2%）	7 (4.1%）	0.322	0.485
Ocular lesions	4 (1.2%)	1 (1.1%）	0 (0%）	3 (1.7%）	-	-
Liver and biliary diseases	67 (20.8%)	15 (16.5%）	17 (29.3%）	35 (20.2%）	3.612	0.353
Thromboembolic diseases	4 (1.2%)	1 (1.1%）	1 (1.7%）	2 (1.2%）	-	-
Complication						
Intestinal perforation	5 (1.6%)	1 (1.1%）	0 (0%）	4 (2.3%）	-	-
Intraepithelial neoplasia	8 (2.5%)	3 (3.3%）	3 (5.2%）	2 (1.2%）	3.238	0.151
Canceration	2 (0.6%)	0 (0%）	0 (0%）	2 (1.2%）	-	-

^*a*^ Comparison between the three groups, *P<0.05; -: not tested for cells containing observed frequencies of 1 or 0.

### Hematology index analysis of UC with different types of *H. pylori* infection

In hematology index, there was no statistical significance in WBC, PLT, NE, LY, MO, EO, BA, TBIL, DBIL, IBIL, TP, GLO, AST, AST/ALT, K, Na, and CL in different types of *H. pylori* infection groups (*P* > 0.05). Inter-group differential analysis demonstrates that the levels of RBC, HGB, PCV, ALB, A/G, and ALT were significantly higher in the type I *H. pylori* infection group compared with both type II *H. pylori* infection group and *H. pylori*-negative group (*P* < 0.05). Conversely, the levels of D-Dimer, CRP, and ESR were significantly lower in the type I *H. pylori* infection group compared with both type II *H. pylori* infection group and *H. pylori*-negative group (*P* < 0.05) ( Table 3).

**TABLE 3:** Hematology index of UC with different types of *H. pylori* infection

Hematology index	Unit	Total (*n* = 322)	Type I *H*. *pylori* infection group (*n* = 91）	Type II *H*. *pylori* infection group (*n* = 58）	*H. pylori*-negative group (*n* = 173）	Statistical value (*χ^2^/F*）	*P* value^*a*^
WBC	×10^12^/L	6.53 (5.3,8.22)	6.33 (5.21,7.44)	7.61 (5.19,8.84)	6.62 (5.39,8.14)	5.407	0.067
RBC	×10^9^/L	4.39 ± 0.58	4.53 ± 0.54	4.26 ± 0.54	4.37 ± 0.6	4.374	0.013*
HGB	g/L	129 (122,143)	132 (208.5,318.5)	125.5 (222,358)	128.5 (228.5,347.5)	6.042	0.049*
PLT	10^9^/L	271 (222,340)	268 (208.5,318.5)	275.5 (222,358)	270.5 (228.5,347.5)	2.493	0.287
PCV	-	0.394 (0.347,0.432)	0.404 (0.337,0.44)	0.375 (0.339,0.418)	0.39 (0.34,0.429)	6.244	0.044*
NE	×10^9^/L	4.05 (3.09,5.42)	3.89 (3.07,4.48）	4.63 (3.15,6.09）	4.15 (3.1,5.5）	4.901	0.086
LY	×10^9^/L	1.77 (1.37,2.11)	1.77 (1.39,2.17）	1.84 (1.57,2.19）	1.74 (1.35,2.08）	1.816	0.403
MO	×10^9^/L	0.42 (0.33,0.56)	0.4 (0.29,0.52）	0.43 (0.34,0.57）	0.42 (0.34,0.57）	4.046	0.132
EO	×10^9^/L	0.15 (0.07,0.26)	0.15 (0.08,0.26）	0.11 (0.06,0.29）	0.15 (0.07,0.25）	0.799	0.67
BA	×10^9^/L	0.02 (0.02,0.04)	0.03 (0.01,0.04）	0.02 (0.02,0.04）	0.02 (0.02,0.04）	0.104	0.95
TBIL	μmol/L	10.2 (7.18,14.93)	10.9 (7.69,15.39)	8.85 (5.9,14.7)	10.24 (7.37,14.8)	4.064	0.131
DBIL	μmol/L	2.6 (0.3,4.72)	1.91 (0.3,4.65)	2.46 (0.3,3.7)	2.63 (0.3,4.9)	1.425	0.49
IBIL	μmol/L	5.9 (3.66,9.29)	6.15 (3.72,10.08)	4.76 (3.11,8.55)	5.97 (3.9,9.07)	3.73	0.155
TP	g/L	68.75 (64.7,73.3)	69.6 (66,73.95)	67.9 (63.04,72.8)	68.2 (64.7,73.1)	3.059	0.217
ALB	g/L	39.5 (35.7,43.18)	40.3 (37.8,43.45)	38.55 (33.9,41.7)	38.9 (35.1,42.77)	7.839	0.02*
GLO	g/L	29.5 (26.6,33)	28.8 (26.4,32.69)	30.04 (27.6,33)	29.7 (26.7,33)	1.693	0.429
A/G	-	1.328 ± 0.302	1.422 ± 0.292	1.265 ± 0.346	1.299 ± 0.281	6.7	0.001*
ALT	U/L	18 (12,26)	20(14,28)	14.04 (11, 24)	17.88(12,26)	6.114	0.047*
AST	U/L	19 (15, 24)	21(16,25)	17.5(14,23)	19(14.43,24)	5.202	0.074
AST/ALT	-	1.23 (0.84,1.37)	1.05 (0.86,1.28)	1.18 (0.84,1.39)	1.14 (0.82,1.38)	2.531	0.282
K	mmol/L	3.82 (3.53,4.06)	3.89 (3.61,4.15)	3.77 (3.58,4)	3.8 (3.51,4.05)	5.473	0.065
Na	mmol/L	140.95 (139,143)	141 (139.85,143)	140.65 (138.43,142)	140.9 (138.6,143)	2.854	0.24
CL	mmol/L	105.6 (103.2,107.3)	105.6 (104,107.8)	105.9 (104,107)	105 (103,107.1)	1.888	0.389
D-Dimer	mg/L	0.33 (0.19,0.67)	0.28 (0.18,0.42)	0.45 (0.19,0.84)	0.38 (0.22,0.78)	9.235	0.01*
CRP	mg/L	5.4 (2.38,14.94)	3.07 (1.63,9.12)	7.57 (2.23,13.6)	6.37 (2.5,22.74)	8.865	0.012*
ESR	MM/H	20(10,38)	13 (6, 18)	26(10.5,42)	24(12,40)	20.682	0.000*
hs-CRP	mg/L	4.69 (1.14,17.37)	2.76 (1.3,6.25)	5.57 (1.44,15.68)	5.66 (0.99,23.72)	2.378	0.305

^*a*^ Comparison between the three groups, *P<0.05.

## DISCUSSION

UC is a chronic, non-specific inflammatory bowel disease characterized by recurrent episodes and delayed healing, accompanied by various extraintestinal manifestations. It significantly impacts the patients’ quality of life and exhibits a propensity for carcinogenesis (10). The etiology of UC remains elusive. Current research findings indicate that the pathogenesis of UC is associated with defects in the intestinal mucosal barrier, genetic susceptibility, microbial and environmental factors, as well as dysregulation of intestinal immune function (11). A meta-analysis revealed an increased risk of UC associated with urban residency, antibiotic exposure, oral contraceptive usage, consumption of carbonated beverages, vitamin D deficiency, and infection with non-*H*. *pylori*-like enterohepatic *Helicobacter* species. Conversely, reduced risks for UC were observed in individuals who had been breastfed, consumed tea, high levels of folate, or were infected with *H. pylori* (12). A systematic review and meta-analysis of 58 studies conducted in 2021 provided robust evidence supporting a protective role of *H. pylori* in UC, with the epidemiological data demonstrating moderate-to-high strength (13).

The prevalence of *H. pylori* exhibits an inverse correlation with UC, suggesting a protective effect of *H. pylori* against the development of UC. Moreover, the eradication of *H. pylori* may result in the recurrence of UC (14). The present study found that the average age of onset of patients with new-onset UC was about 50 years old, the disease was mainly mild, and the lesion was extensive in the colon. The predominant form of UC observed in previous studies is the chronic recurrent type, characterized by extensive involvement of the left hemicolon and colon, which aligns with the findings reported in this study (15). In this study, the patients with UC in the *H. pylori*-negative group exhibited a protracted disease course, a high recurrence rate, and severe disease severity. Based on the analysis of clinical characteristics, it can be considered that *H. pylori* confers a protective effect against UC.

*H. pylori* is a highly diverse bacterium, exhibiting a wide array of virulence factors that have been characterized. The main virulence factors encompass adherence (BabA, HopZ, SabA), effector delivery (CagA, Type IV), motility (Flagella, Flagellar glycosylation), secretion systems, exotoxin (VacA), immune modulation (HP-NAP, Lewis antigen, LPS, OipA), stress survival (urease), etc. *CagA* and *VacA* genes have been extensively investigated as virulence markers of *H. pylori*. The presence of *CagA* and *VacA* genes in *H. pylori* strains is closely associated with the occurrence and progression of various gastric diseases (16, 17).

The virulence factors of *H. pylori* not only participate in the initiation of inflammatory responses but also exert control and regulation over these responses, thereby sustaining chronic inflammation, and the most important is the interaction between bacterial virulence factors and host-microecology (4). *H. pylori* colonization resulted in a reduction of Th17 cells and downregulation of mRNA levels for IL-17A, IL-17F, and IL-21 in the colon. Meanwhile, *H. pylori* colonization led to an increase in Treg cells and upregulation of IL-10 expression (18). Serum exosomes derived from patients with *H. pylori*-positive gastritis can attenuate the expression of MCP-1 and MIP-1α in intestinal epithelial cells via the NLRP12-Notch signaling pathway, thereby ameliorating dextran sodium sulfate-induced colitis (19). The virulence factors of *H. pylori* also play crucial roles in UC. In CagA transgenic mice, CagA-mediated IκB exacerbates DSS-induced colitis by reducing the threshold for NF-κB activation. Additionally, CagA activates inflammasome independently of NF-κB signaling, thereby further enhancing inflammation. At the transcriptional level, the recovery of colitis-injured mucosa *in vitro* is impeded by upregulated Claudin-2 expression facilitated through a CDX2-dependent mechanism (20, 21). The findings of this study demonstrate that infection with highly virulent type I *H. pylori* is associated with a milder form of UC in terms of disease severity. Moreover, extraintestinal manifestations primarily manifest as liver and biliary diseases, joint lesions, and cutaneous mucosal manifestations. Notably, the incidence of joint lesions is low in cases of type I *H. pylori* infection. These observations suggest that the virulence factor of *H. pylori* exerts a protective effect on the severity of UC and joint lesions; however, the protective ability conferred by type II *H. pylori* appears to be less significant.

Hematology index not only serve as a supplementary diagnostic tool for UC but also facilitate the evaluation of the condition in cases of chronic recurrent UC (22). This study conducted a comprehensive analysis of the blood routine, biochemical, and other laboratory indicators in patients with UC. However, only representative indicators were presented in the results. The results revealed that the levels of RBC, HGB, PCV, ALB, A/G, and ALT were significantly elevated in patients with type I *H. pylori* infection compared with those with type II *H. pylori* infection and *H. pylori*-negative group. Additionally, the levels of D-Dimer, CRP, and ESR were significantly lower than those in type II *H. pylori* infection group and *H. pylori*-negative group. These hematologic indices changes suggest that UC patients with type I *H. pylori* infection exhibit reduced incidences of anemia, chronic liver disease, thrombosis, and inflammation. Therefore, the highly virulent strain of *H. pylori* (CagA/VacA) may potentially mitigate the complications and disease severity associated with UC, whereas the impact of type II *H. pylori* on ameliorating these complications appears to be limited.

Currently, numerous *H. pylori*-related guidelines and consensus recommend universal eradication of *H. pylori*; however, the objective of achieving universal eradication is hindered by public health pressures in several countries (23, 24). The eradication of *H. pylori* remains a subject of controversy in the context of inflammatory bowel disease, gastroesophageal reflux disease, asthma, and other diseases (5). In conclusion, the virulence of *H. pylori* plays a crucial role in protecting against UC, and infection with hypervirulent strains may improve disease severity and the occurrence of complications. Consequently, virulence factors should be taken into consideration when targeting *H. pylori* eradication in clinical practice, particularly in UC patients. It is crucial to evaluate the individual benefits to optimize personalized eradication therapies.

## Data Availability

The data supporting the conclusions of this article has been shown without undue reservation. The raw data relating to patients cannot be shared in compliance with agreements with our partners (People’s Hospital of Xinjiang Uygur Autonomous and YIDUCLOUD data platform). However, other parties can apply to People’s Hospital of Xinjiang Uygur Autonomous and YIDUCLOUD data platform for research data access.

## References

[1] Quansah E, Gardey E, Ramoji A, Meyer-Zedler T, Goehrig B, Heutelbeck A, Hoeppener S, Schmitt M, Waldner M, Stallmach A, Popp J. 2023. Intestinal epithelial barrier integrity investigated by label-free techniques in ulcerative colitis patients. Sci Rep 13:2681. doi:10.1038/s41598-023-29649-y 36792686 PMC9931702

[2] Li M-X, Li M-Y, Lei J-X, Wu Y-Z, Li Z-H, Chen L-M, Zhou C-L, Su J-Y, Huang G-X, Huang X-Q, Zheng X-B. 2022. Huangqin decoction ameliorates DSS-induced ulcerative colitis: role of gut microbiota and amino acid metabolism, mTOR pathway and intestinal epithelial barrier. Phytomedicine 100:154052. doi:10.1016/j.phymed.2022.154052 35344714

[3] Kahlam A, Khrais A, Khalessi A, Ahlawat S. 2023. Trends and complication rates in ulcerative colitis patients with and without Helicobacter pylori infections. Cureus 15:e37345. doi:10.7759/cureus.37345 37182047 PMC10169286

[4] Tanner S, Katz J, Cominelli F, Regueiro M, Cooper G, Mansoor E. 2023. Inflammatory bowel disease and Helicobacter pylori: protective or present? Inflamm Bowel Dis 29:1005–1007. doi:10.1093/ibd/izac202 36169397

[5] He J, Liu Y, Ouyang Q, Li R, Li J, Chen W, Hu W, He L, Bao Q, Li P, Hu C. 2022. Helicobacter pylori and unignorable extragastric diseases: mechanism and implications. Front Microbiol 13:972777. doi:10.3389/fmicb.2022.972777 35992650 PMC9386483

[6] Qin C, Huang GR, Guan AX, Zhou WT, Chen H, Luo PP, Luo XK, Huang YQ, Huang ZS. 2024. Mechanistic research: selenium regulates virulence factors, reducing adhesion ability and inflammatory damage of Helicobacter pylori. World J Gastroenterol 30:91–107. doi:10.3748/wjg.v30.i1.91 38293320 PMC10823904

[7] Baj J, Forma A, Sitarz M, Portincasa P, Garruti G, Krasowska D, Maciejewski R. 2020. Helicobacter pylori virulence factors- mechanisms of bacterial pathogenicity in the gastric microenvironment. Cells 10:27. doi:10.3390/cells10010027 33375694 PMC7824444

[8] Liu W, Kong W, Hui W, Wang C, Jiang Q, Shi H, Gao F. 2022. Characteristics of different types of Helicobacter pylori: new evidence from non-amplified white light endoscopy. Front Microbiol 13:999564. doi:10.3389/fmicb.2022.999564 36713187 PMC9881747

[9] Inflammatory Bowel Disease Group, Chinese Society of Gastroenterology, Chinese Medical Association. 2018. Chinese consensus on diagnosis and treatment in inflammatory bowel disease (2018, Beijing). Chinese Journal of Inflammatory Bowel Diseases 2:173–190. doi:10.1111/1751-2980.12994

[10] Yao D, Dong M, Dai C, Wu S. 2019. Inflammation and inflammatory cytokine contribute to the initiation and development of ulcerative colitis and its associated cancer. Inflamm Bowel Dis 25:1595–1602. doi:10.1093/ibd/izz149 31287863

[11] Ungaro R, Mehandru S, Allen PB, Peyrin-Biroulet L, Colombel J-F. 2017. Ulcerative colitis. Lancet 389:1756–1770. doi:10.1016/S0140-6736(16)32126-2 27914657 PMC6487890

[12] Piovani D, Danese S, Peyrin-Biroulet L, Nikolopoulos GK, Lytras T, Bonovas S. 2019. Environmental risk factors for inflammatory bowel diseases: an umbrella review of meta-analyses. Gastroenterology 157:647–659. doi:10.1053/j.gastro.2019.04.016 31014995

[13] Shirzad-Aski H, Besharat S, Kienesberger S, Sohrabi A, Roshandel G, Amiriani T, Norouzi A, Keshtkar A. 2021. Association between Helicobacter pylori colonization and inflammatory bowel disease: a systematic review and meta-analysis. J Clin Gastroenterol 55:380–392. doi:10.1097/MCG.0000000000001415 32833699

[14] Zhong Y, Zhang Z, Lin Y, Wu L. 2021. The relationship between Helicobacter pylori and inflammatory bowel disease. Arch Iran Med 24:317–325. doi:10.34172/aim.2021.44 34196192

[15] Shao S, Huang M, Zhang H, Peng G, Song M, Liu J, Xu D. 2022. A retrospective analysis of clinical features and treatment of the inflammatory bowel disease in China. J Inflamm Res 15:3587–3597. doi:10.2147/JIR.S353329 35757458 PMC9231537

[16] Lee DH, Ha JH, Shin JI, et al.. 2021. Increased risk of severe gastric symptoms by virulence factors vacAs1c, alpA, babA2, and hopZ in Helicobacter pylori infection. J Microbiol Biotechnol 31:368–379. doi:10.4014/jmb.2101.01023 33622995 PMC9705970

[17] Barreyro FJ, Sanchez N, Caronia MV, Elizondo K, Jordá G, Schneider A, Zapata PD. 2023. Low-grade duodenal eosinophilia is associated with cagA in Helicobacter pylori-related dyspepsia. J Gastroenterol Hepatol 38:274–282. doi:10.1111/jgh.16051 36334009

[18] Zhang H, Dai Y, Liu Y, Wu T, Li J, Wang X, Wang W. 2018. Helicobacter pylori colonization protects against chronic experimental colitis by regulating Th17/Treg balance. Inflamm Bowel Dis 24:1481–1492. doi:10.1093/ibd/izy107 29788098

[19] Chen Y, Huang J, Li H, Li P, Xu C. 2020. Serum exosomes derived from Hp-positive gastritis patients inhibit MCP-1 and MIP-1α expression via NLRP12-Notch signaling pathway in intestinal epithelial cells and improve DSS-induced colitis in mice. Int Immunopharmacol 88:107012. doi:10.1016/j.intimp.2020.107012 33182033

[20] Suzuki N, Murata-Kamiya N, Yanagiya K, Suda W, Hattori M, Kanda H, Bingo A, Fujii Y, Maeda S, Koike K, Hatakeyama M. 2015. Mutual reinforcement of inflammation and carcinogenesis by the Helicobacter pylori CagA oncoprotein. Sci Rep 5:10024. doi:10.1038/srep10024 25944120 PMC4421872

[21] Guo Y, Xu C, Gong R, Hu T, Zhang X, Xie X, Chi J, Li H, Xia X, Liu X. 2022. Exosomal CagA from Helicobacter pylori aggravates intestinal epithelium barrier dysfunction in chronic colitis by facilitating Claudin-2 expression. Gut Pathog 14:13. doi:10.1186/s13099-022-00486-0 35331316 PMC8944046

[22] Feng W, Liu Y, Zhu L, Xu L, Shen H. 2022. Evaluation of neutrophil-to-lymphocyte ratio and platelet-to-lymphocyte ratio as potential markers for ulcerative colitis: a retrospective study. BMC Gastroenterol 22:485. doi:10.1186/s12876-022-02571-9 36424535 PMC9685881

[23] Malfertheiner P, Megraud F, Rokkas T, Gisbert JP, Liou J-M, Schulz C, Gasbarrini A, Hunt RH, Leja M, O’Morain C, Rugge M, Suerbaum S, Tilg H, Sugano K, El-Omar EM, European Helicobacter and Microbiota Study group. 2022. Management of Helicobacter pylori infection: the Maastricht VI/Florence consensus report. Gut:gutjnl-2022-327745. doi:10.1136/gutjnl-2022-327745 35944925

[24] Zhou L, Lu H, Song Z, Lyu B, Chen Y, Wang J, Xia J, Zhao Z. 2022. Chinese national clinical practice guideline on Helicobacter pylori eradication treatment. Chin Med J (Engl) 135:2899–2910. doi:10.1097/CM9.0000000000002546 36579940 PMC10106216

